# HTLV-1-Derived Exosomes Drive Transcriptional Reprogramming of Monocytes Toward a Mixed M1/M2 Phenotype in HAM/TSP

**DOI:** 10.3390/pathogens15070704

**Published:** 2026-07-03

**Authors:** Catherine A. MacNary, Sai Chaitanya Rajendra Gaekwar, Alexander Lemenze, Ayaan Naik, Ritesh Tandon, Salwa Ahmed, Bobby Brooke Herrera, Pooja Jain

**Affiliations:** 1 Department of Microbiology & Immunology, Drexel University College of Medicine, Philadelphia, PA 19129, USAayaanshivnaik@gmail.com (A.N.);; 2 Molecular and Genomics Informatics Core, Rutgers New Jersey Medical School, Newark, NJ 07103, USA; 3 Department of Medicine, Division of Allergy, Immunology, and Infectious Diseases, Rutgers Global Health Institute, New Brunswick, NJ 08901, USA; 4 Child Health Institute of New Jersey, Rutgers Robert Wood Johnson Medical School, Rutgers University, New Brunswick, NJ 08901, USA

**Keywords:** HTLV-1, HAM/TSP, monocytes, exosomes

## Abstract

Human T-lymphotropic virus type 1 (HTLV-1)-associated myelopathy/tropical spastic paraparesis (HAM/TSP) is a chronic neuroinflammatory disorder often leading to demyelination of the spinal cord. Progression to HAM/TSP is closely associated with the high proviral load and the presence of virally infected CD4+ T cells that release extracellular vesicles (EVs). Exosomes, an EV subtype released by many cell types, transport proteins and nucleic acids that regulate intercellular communication and have been implicated in the progression of cancer and neuroinflammatory diseases. Herein, we have studied the effect of exosomes from HTLV-1 infected cells on the Peripheral Blood Mononuclear Cells (PBMCs) of HAM/TSP patients by single-cell sequencing utilizing innovative Honeycomb technology. We observed a distinct transcriptional response in monocyte populations compared with other immune cell types. Given that monocytes remain understudied in HTLV-1 pathogenesis, these findings highlight a potential role for infection-derived exosomes in shaping monocyte-driven immune dysregulation in HAM/TSP. A total of 41 genes were identified to be differentially expressed in HAM/TSP monocytes treated with exosomes; 28 were upregulated and 13 were downregulated. The most significantly altered genes are involved in chemokine activity and signaling, macrophage differentiation, lipid metabolism, and lysosomal function. Overall, our data suggests that exosome-treated HAM/TSP monocytes undergo immune remodeling that favors cell recruitment, activation, and a shift toward a mixed M1/M2-like phenotype. Such a shift may support viral persistence and chronic inflammation. These findings highlight a potential therapeutic pathway for addressing HTLV-1-induced neuroinflammation by modulating exosome-mediated signaling.

## 1. Introduction

HTLV-1 is a retrovirus infecting an estimated 5–10 million individuals globally and is the etiologic agent of both HAM/TSP and adult T cell leukemia/lymphoma (ATLL). HAM/TSP is a progressive neuroinflammatory disorder characterized by immune-mediated central nervous system (CNS) damage [1,2]. The pathogenesis of HAM/TSP is thought to involve chronic immune activation, particularly through infiltration of HTLV-1-infected CD4^+^ T cells and activated cytotoxic CD8^+^ T cells, alongside monocyte and macrophage dysregulation in peripheral and CNS compartments [3,4,5]. Despite this, monocytes remain among the least characterized immune cell populations in HTLV-1 pathogenesis, and their potential contribution to immune dysregulation and neuroinflammation in HAM/TSP remains poorly understood.

Monocytes are large, single-lobed white blood cells that operate as part of the innate immune system to monitor the body for pathogens and coordinate the immune response during times of infection and inflammation [6]. There are three subtypes of monocytes; Classical (CD14^+^CD16^−^) which circulate in the blood, Intermediate (CD14^+^CD16^+^) which are transitional and proinflammatory, and Nonclassical (CD14^−^CD16^+^) which patrols the vascular epithelium [7]. Classical monocytes are antigen presenting, phagocytic cells capable of differentiating into several peripheral cell types essential for host defense. Macrophages are essential for clearing of pathogens and cellular debris, while dendritic cells patrol peripheral tissues (e.g., skin) and present antigens to activate T cells [8]. Monocytes can differentiate into macrophages that exhibit a spectrum of activation states. Classically activated M1 macrophages are generally associated with pro-inflammatory cytokine production, antimicrobial responses, and tissue inflammation; whereas alternatively activated M2 macrophages are linked to immune regulation, tissue repair, and anti-inflammatory functions. Although macrophage polarization exists along a continuum, the M1/M2 framework remains useful for describing broad functional phenotypes [9,10]. Monocytes are also essential mediators of HIV pathogenesis by acting as a reservoir and facilitating viral spread early in infection [11]. Though HIV and HTLV-1 share many similarities, the role of monocyte in HTLV-1 pathogenesis remains underexplored [12]. In both HTLV-1 and HIV, Intermediate monocytes (CD14^+^CD16^+^) have been implicated in disease progression which tend to skew towards a more immunoregulatory M2 profile [13,14].

Extracellular vesicles (EVs) are lipid-bound particles released into the extracellular environment that carry lipids, proteins, and nucleic acids. EVs include apoptotic bodies, microvesicles, and exosomes, the latter being the focus of this study [15]. Exosomes, or intraluminal vesicles (30–150 nm), are key mediators of cell–cell communication and immune modulation. Exosomes have been found to potentially contain pathogenic proteins within the CNS which could aid in progression of neuro-inflammatory diseases like Alzheimer’s [16,17]. This suggests that exosomes may also play a role in HAM/TSP pathogenesis within the spinal cord. Exosomes have also recently emerged as a key area of investigation in cancer research, with growing evidence linking exosome and immune checkpoint protein (ICP) expressions to poor prognosis in Melanoma, Non-Small Cell Lung Cancer (NSCLC), and Ovarian Cancer [18,19]. In response, multiple clinical trials have been launched to evaluate the effectiveness of combining exosome inhibitors with standard therapies. Preliminary results suggest that blocking exosome signaling can enhance treatment outcomes by slowing disease progression [20]. This approach represents a promising avenue for next-generation cancer therapies and may hold similar potential for other progressive diseases in which exosomes play a critical role; particularly neuroinflammatory and neurodegenerative conditions such as HAM/TSP.

Recent studies have shown that HTLV-1-infected cells secrete exosomes, containing viral proteins such as Tax, viral transcripts like HBZ, and immunomodulatory host factors [18,19]. The HTLV-1 virus immunomodulates T cells using exosomes which, along with ICPs, leads to the development of neuroinflammation [21]. Exosomes help enhance HTLV-1 infection through polarizing CD4^+^ cells and reducing the effect of CD8^+^ cells [9]. Exosomes can be taken up by recipient cells and induce activation of survival and inflammatory pathways, including NF-κB, AKT, and mTOR, thereby supporting a proinflammatory microenvironment and promoting viral persistence [18]. In addition to stimulating inflammation, HTLV-1-derived exosomes have been shown to alter immune cell polarization [22]. While HAM/TSP is associated with a dominant M1-like macrophage phenotype, recent data suggest that exosomes may drive M2-like polarization by upregulating markers such as IL10, MRC1 (CD206), PPARG, and CSF1 [22]. These M2-associated genes contribute to immune modulation, tissue remodeling, and chronic inflammation, potentially facilitating viral immune evasion and perpetuating the neuropathology observed in HAM/TSP. In this study, we observed a distinct response in monocytes of HAM/TSP patient upon exposure to HTLV-1 exposed exosomes.

The direct impact of HTLV-1-derived exosomes on monocyte/macrophage gene expression and polarization dynamics in the context of HAM/TSP remains underexplored. As exosomes contribute to cell-to-cell contact of uninfected cells, inflammatory cytokine release, and severity of HAM/TSP, understanding their effect on gene expression remains crucial for elucidating HTLV immunopathogenicity. To achieve this, we analyzed the transcriptional response of HAM/TSP patient PBMCs following treatment with HTLV-1-derived exosomes, with a focus on pathways involved in macrophage polarization and immune modulation. The HIVE CLX system utilized further captures cell heterogeneity commonly hidden by traditional “bulk” in RNA sequencing. Herein we show that macrophages seem to experience the most dysregulation when infected by HTLV-1, and that exosome-treated monocytes exhibit gene expression that enables M2-like immunoregulation. This research showcases how these genetic shifts favor viral escape, continued inflammation, and greater viral spread in HTLV-1, allowing further exploration into therapeutic targets in the future.

## 2. Methods

### 2.1. Exosome Preparation and Quantification

MT-4 cells were selected as the source of HTLV-1-derived exosomes because they are a well-characterized HTLV-1-producing T cell line that expresses high levels of viral proteins and have been widely used to study HTLV-1-associated extracellular vesicles. HTLV-1-infected MT-4 cells were cultured in complete RPMI media supplemented with 10% exosome-depleted fetal bovine serum (FBS). Cells were seeded at a density of 3–5 × 10^5^ cells/mL and maintained at 37 °C in a humidified 5% CO_2_ incubator. Culture supernatants were collected after 72 h. To remove cells and debris, supernatants were subjected to sequential centrifugation at 400× *g* and 2000× *g* for 10 min each. The clarified supernatant was then filtered through a 0.45 µm membrane filter to eliminate residual particles. Exosomes were isolated by ultracentrifugation at 100,000× *g* for 2 h at 4 °C. The resulting pellet was washed in Dulbecco’s phosphate-buffered saline (DPBS) and centrifuged again at 100,000× *g* for 75 min at 4 °C. The final exosome pellet was resuspended in DPBS and stored at −80 °C until further use.

Exosome protein concentration was determined using the Pierce™ BCA Protein Assay Kit (Thermo Fisher Scientific, Waltham, MA, USA) according to the manufacturer’s instructions [21]. Exosome preparations were characterized using nanoparticle tracking analysis and exosome-associated markers as previously described to confirm enrichment of extracellular vesicles prior to downstream experiments [15]. Jurkat cell-derived exosomes were isolated and characterized using the same protocol and served as the control exosome preparation throughout the study.

### 2.2. Honeycomb Single-Cell Sequencing

HAM/TSP PBMCs from a HOST (HTLV Outcomes Study, [23,24]) participant were utilized for the comparative single-cell RNA sequencing (ScRNA-seq) to assess the effects of HTLV-derived exosomes on various cell types. PBMCs were thawed, washed, and resuspended in phosphate-buffered saline (PBS) supplemented with 1% bovine serum albumin (BSA). Cell counts and viability were determined using a Countess 3 automated cell counter (Thermo Fisher Scientific). A total of 2.5 × 10^5^ PBMCs were divided into two equal groups. One group, serving as a control, was treated with 30 μg of Jurkat-derived exosomes, while the second group was treated with 30 µg of HTLV-1-derived exosomes. Cells were incubated for 18 h at 37 °C in a humidified 5% CO_2_ incubator. Following incubation, approximately 20,000 cells per condition were loaded into HIVE CLX collectors according to the manufacturer’s instructions (Honeycomb Bio, Waltham, MA, USA). The loaded collectors were stored at −80 °C until downstream processing. Single-cell RNA sequencing (scRNA-seq) was performed using the HIVE CLX system (Honeycomb Bio) following the manufacturer’s protocol.

### 2.3. Primary Analysis of the Data and Quality Control

Raw scRNA-seq data generated from the HIVE CLX platform were processed and analyzed using the Seurat v4.0 R package. Initial quality control filtering was performed to exclude low-quality or potentially artifactual cells. Cells expressing fewer than 100 genes or more than 4500 genes were excluded to remove empty droplets and potential multiplets, respectively. Additionally, cells with >10% mitochondrial gene expression, >45% ribosomal gene content, or >20% hemoglobin gene expression were removed to eliminate stress, dying, or contaminating cells. Following filtering, gene expression matrices were normalized and scaled using Seurat’s standard workflow. Principal Component Analysis (PCA) was applied for dimensionality reduction, and the most significant principal components were used for downstream clustering. Cell populations were visualized using Uniform Manifold Approximation and Projection (UMAP). Cell populations were annotated using the Honeycomb bioinformatics pipeline and confirmed based on canonical lineage markers. T cells were identified by expression of CD3 together with CD4 or CD8 markers, B cells by CD19 and CD20 expression, monocytes by CD14 expression, dendritic cells by CD11c expression, and NK cells by expression of CD56 and CD16 in the absence of CD3 expression. Monocyte clusters were subsequently utilized for the downstream comparative analyses. Differential gene expression analysis between exosome-treated and untreated samples was performed using the Wilcoxon rank-sum test, with significance thresholds set at a false discovery rate (FDR) < 0.05 and an absolute log_2_ fold change > 1.

### 2.4. Ingenuity Pathway Analysis (IPA)

To interpret the biological significance of differentially expressed genes (DEGs) identified in monocytes, Ingenuity Pathway Analysis (IPA, Qiagen, Redwood City, CA, USA) was employed. Given that gene-level changes alone do not fully capture functional cellular responses, IPA was used to contextualize transcriptional alterations within known signaling pathways, molecular networks, and regulatory interactions, particularly those relevant to immune modulation and neuroinflammation. Significantly upregulated and downregulated genes meeting the defined statistical thresholds (FDR < 0.05, |log_2_FC| > 1) were imported into the IPA platform. Core analysis was performed to identify enriched canonical pathways, upstream regulators, and functional networks associated with the observed transcriptional changes. Pathway enrichment was prioritized based on relevance to monocyte biology, immune activation, and inflammatory signaling, including pathways related to macrophage polarization, cytokine signaling, and leukocyte trafficking. IPA-generated networks were used to visualize interactions among DEGs and to predict activation or inhibition states of key signaling pathways.

## 3. Results

### 3.1. Quality Control and Cell-Type Annotation of Single-Cell RNA-Seq Data

To ensure high-quality single-cell transcriptomic analysis, stringent quality control (QC) filtering was applied to the scRNA-seq dataset. Cells with low gene counts, high mitochondrial gene expression, or excessive ribosomal and hemoglobin gene content were excluded, resulting in a refined dataset suitable for downstream analysis. Cell numbers for each annotated immune-cell population are provided in Supplementary Figure S1D. Monocytes constituted the largest population analyzed, with 149 cells identified in the Jurkat-derived control exosome condition and 172 cells in the HTLV-1-derived exosome condition. Following QC, dimensionality reduction and clustering were performed, and cell populations were annotated using canonical marker genes and SingleR-based classification. Distinct immune cell populations-including CD4^+^ and CD8^+^ T cells, B cells, monocytes, and dendritic cells-were clearly identified (Supplementary Figure S1A,B). Comparative analysis of cell-type composition revealed shifts in immune cell proportions between control and HTLV-1-derived exosome-treated conditions, including a relative decrease in monocyte populations (Supplementary Figure S1C), suggesting that HTLV-1-derived exosomes may influence immune cell distribution. The entire data set has been deposited in the NCBI library with the accession number GSE308157.

### 3.2. Monocytes Exhibit the Most Pronounced Transcriptional Response to HTLV-1-Derived Exosomes

**Figure 1:**
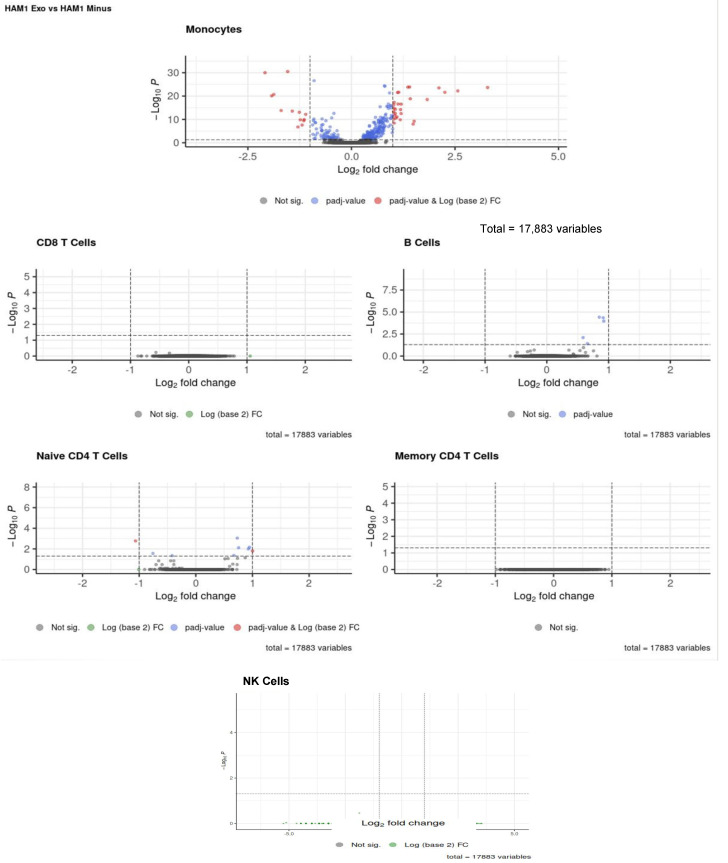
**Differential gene expression across immune cell types in HAM/TSP PBMC specimen treated with Jurkat-derived exosomes (control) or HTLV-1-derived exosomes.** Volcano plots showing differentially expressed genes (DEGs) in monocytes, CD8 T cells, B cells, naive CD4 T cells, and memory CD4 T cells comparing HAM/TSP patients without and with exosome treatment. The *x*-axis shows Log2 foldchange, and the *y*-axis demonstrates a log_10_ adjusted *p*-value. The red dots represent genes that were significantly differentially expressed by fold change and statistical significance (|log_2_FC| > 1 and *p*adj < 0.05). The blue dots represent genes that were significant by adjusted *p*-value and not significant fold change. The green dots represent genes that were significant by fold change only. Of the immune cell subsets, only monocytes showed significant changes in transcriptionally altered genes. A total of 17,883 genes were analyzed per cell type.

To identify cell populations most affected by exosome exposure, differential gene expression analysis was performed across major immune cell subsets. Among all analyzed cell types, monocytes exhibited the most substantial transcriptional changes in response to HTLV-1-derived exosomes (Figure 1). A total of 41 genes were significantly differentially expressed in monocytes, with 28 genes upregulated and 13 genes downregulated (Table 1). These genes met the defined statistical thresholds (FDR < 0.05, |log_2_FC| > 1), indicating robust transcriptional remodeling in this cell population. The magnitude of transcriptional changes varied across genes. Highly upregulated genes included CXCL5, PPBP, and IL7R, while strongly downregulated genes included FUCA1 and APOC1, highlighting a broad alteration in inflammatory, metabolic, and immune-related pathways. These findings identify monocytes as the primary responders to HTLV-1-derived exosome exposure and support their potential role in mediating downstream immune dysregulation in HAM/TSP.

### 3.3. Functional Categorization of Differentially Expressed Genes Reveals Immune and Metabolic Pathway Alterations

**Figure 2:**
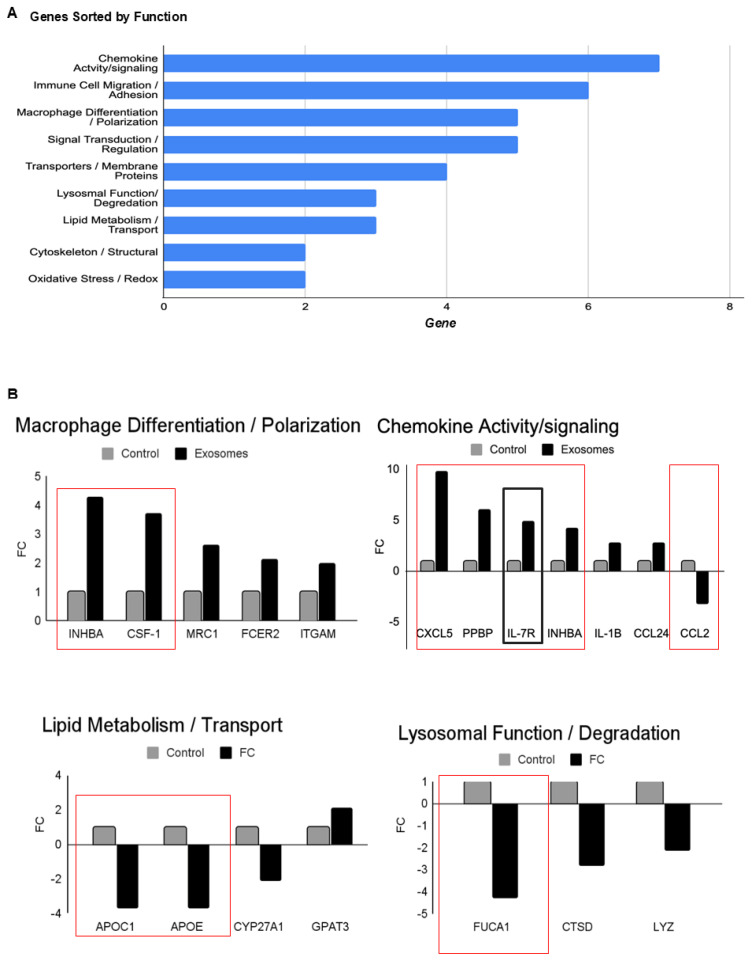
**Graphs displaying data by gene function.** (**A**) genes sorted by function. (**B**) expanded graphs showing each gene sorted by function. Please note there are some genes that fit into more than one category and as such may be seen more than once.

These genes were classified into nine Gene Ontology (GO) Biological Process categories, including: (1) chemokine activity and signaling, (2) immune cell migration and adhesion, (3) macrophage differentiation and polarization, (4) membrane transporters and proteins, (5) signal transduction and regulation, (6) lipid metabolism and transport, (7) lysosomal function and degradation, (8) cytoskeletal and structural organization, and (9) oxidative stress/REDOX processes. Because several genes participate in multiple biological processes, some overlap between categories is expected (Figure 2A).

The genes that have chemokine activity and signaling are CXCL5, PPBP, IL-7R, INHBA, IL-1B, CCL24, and CCL2 (Figure 2B). The most upregulated of these are CXCL5, PPBP, IL-7R, and CCL2. CXCL5 (FC 9.8) is a chemokine that attracts neutrophils. PPBP (FC 6) is a growth factor that acts as a potent chemoattractant and activator of neutrophils. IL-7R (FC 4.9) is the receptor for IL-7 present on T cells. It also plays a role in upregulating anti-apoptotic genes such as the Bcl-2 family proteins. The moderately upregulated of these are INHBA, IL-1β, and CCL24. INHBA (FC 4.3) encodes for inhibin and activin which are both part of the TNF-β pathway [25,26]. IL-1β (FC 2.8) is a proinflammatory cytokine that shifts the immune environment to a more inflammatory M1 phenotype. CCL24 (FC 2.8) is a chemotaxic molecule that has a negative effect on monocyte recruitment. The only downregulated gene was CCL2 (FC 0.31) which also is important for monocyte recruitment.

Overall, genes involved with immune cell migration and adhesion are MMP9, SPN, SDC2, CLEC4A, EMP1, and ITGAM. MMP9 (FC 2.3) degrades extracellular matrix to help immune cells migrate through the tissues and promotes an inflammatory M1 response. SPN (FC 2.3) is a surface protein involved in rolling (initial adhesion to the vascular endothelium) and transmigration through the epithelium, helping with monocyte mobility and immune surveillance. SDC2 (FC 2.3) is a glycoprotein that supports cell adhesion and ECM interaction, increasing migration readiness in monocytes. EMP1 (FC 2.1) is similar as it also plays a role in cell adhesion and migration. ITGAM (FC 2) is involved in monocyte adhesion, migration, and phagocytosis and depending on context can skew the immune environment towards inflammatory or immunoregulatory [6,10]. Overall, we see a marked increase in cell migration and adhesion suggesting that HTLV-1 virus helps monocytes infiltrate tissue and increase chronic inflammation to promote viral spread.

The genes involved in macrophage differentiation and polarization are INHBA, CSF-1, MRC1, FCER2, and ITGAM. As discussed previously, INHBA and ITGAM play powerful roles in chemokine activity and immune cell migration, respectively. As such, they have profound effects on macrophage differentiation as well. INHBA skews more towards M2 and ITGAM can skew towards M1 or M2 depending on context. CSF-1, MRC1, and FCER2 are all part of CSF-1/CSF1R signaling pathway (macrophage colony-stimulating factor pathway) [10,14].

The genes associated with membrane transport are SLC44A1, SLC11A1, KCNK6, and PLXNC1. SLC44A1 (FC 2.1) transports choline for phospholipid synthesis and membrane formation, which is important for vesicle formation. SLC11A1 (FC 2.1) is a transporter that helps bring metals (Fe, etc.) into phagosomes to promote pathogen killing. KCNK6 (FC 2) is a potassium channel that enables K(+) efflux coupled to ATP-induced NLRP3 inflammasome activation upon bacterial infection in macrophages. PLXNC1 (FC 0.5) is a transmembrane receptor for semaphorins, which regulate axon guidance, cell motility and migration, and immune response. By downregulating PLXNC1, HTLV-1 can reduce immune activation and dendritic cross talk. Collectively, these transcriptional changes may contribute to a cellular environment that favors HTLV-1 persistence and immune evasion—except SLC11A1, which may be upregulated by the host in response to other host cell defenses failing due to exosome/viral interference.

Genes associated with signal transduction and regulation include ARRDC4, CDKN1A, YWHAH, FYB1, and ATF5. ARRDC4 (FC 2.6) is a member of the α-arrestin family involved in metabolic stress signaling and regulation of membrane receptor trafficking. Its upregulation may indicate increased cellular stress and could influence receptor recycling or degradation, potentially affecting how monocytes respond to extracellular signals. CDKN1A (FC 2.6), which encodes p21, is a cell cycle inhibitor involved in DNA damage response and regulation of cell proliferation. YWHAH (FC 2.3), a 14-3-3 family protein, acts as a signaling scaffold and plays a role in regulating cell survival, apoptosis, and immune signaling cascades. FYB1 (FC 0.4) is an adaptor protein important in T cell receptor signaling and integrin activation in immune cells; its downregulation may impair immune cell activation or coordination, contributing to immune dysfunction. ATF5 (FC 0.43) is a transcription factor involved in cellular stress responses and survival signaling, and its decreased expression could reflect disrupted stress adaptation or apoptotic priming. Collectively, these transcriptional changes may enhance cellular adaptation to stress and could potentially create conditions favorable for HTLV-1 persistence.

Genes associated with lipid metabolism and transport are APOC1, APOE, CYP27A1, and GPAT3. APOC1 (FC 0.27) and APOE (FC 0.27) are both important in lipid transport, with APOC1 important for breaking down lipids inside of chylomicrons and APOE needed for the degradation of chylomicrons. CYP27A1 (FC 0.47) is an enzyme part of the cytochrome p450 family that helps metabolize drugs and lipids. A reduction in CYP27A1 transcription would likely slow lipid metabolism. GPAT3 (FC 2.1) is an enzyme essential in converting glycerol-3 phosphate to lysophosphatidic acid, a key precursor in the synthesis of triacylglycerol. Overall, monocyte transcription of genes related to lipid metabolism seems to signal greater storage of lipids in cells rather than active use.

Genes associated with lysosomal function and degradation are FUCA1, CTSD, and LYZ. FUCA1 (FC 0.23) is a lysosomal storage enzyme whose role is to break down fructose-containing glycoproteins and glycolipids. CTSD (FC 0.35) is important in protein turnover and cell degradation. Interestingly, downregulation of CTSD seemingly has the opposite effect of the upregulation seen in CDKN1A, as we previously observed. LYZ (FC 0.47) is an enzyme important for destroying certain Gram-positive bacteria [27]. Overall, transcriptional changes in genes related to lysosomes show a decrease in important immune functions.

The smallest categories were for genes associated with cytoskeletal/structural organization and oxidative stress/REDOX processes. Those associated with the former are GSN and SLAMF7. GSN (FC 2.1) functions in both assembly and disassembly of actin filaments helpful for movement throughout the cell. SLAMF7 (FC 0.4) is an important component of the cytoskeleton that helps with phagocytosis and is an upstream effector that stimulates NK cell activation. Genes associated with oxidative stress/REDOX processes are SOD2 and TXNIP. SOD2 (FC 0.43) is a mitochondrial enzyme that converts free radicals into hydrogen peroxide to be used by lysosomes. By decreasing this enzyme, free radicals build up in the cell causing oxidative stress leading to DNA damage. Overall, an increase in oxidative stress is seen in monocytes treated with exosomes.

### 3.4. Gene Ontology Enrichment Highlights Pathways Involved in Inflammation, Migration, and Macrophage Polarization

**Figure 3:**
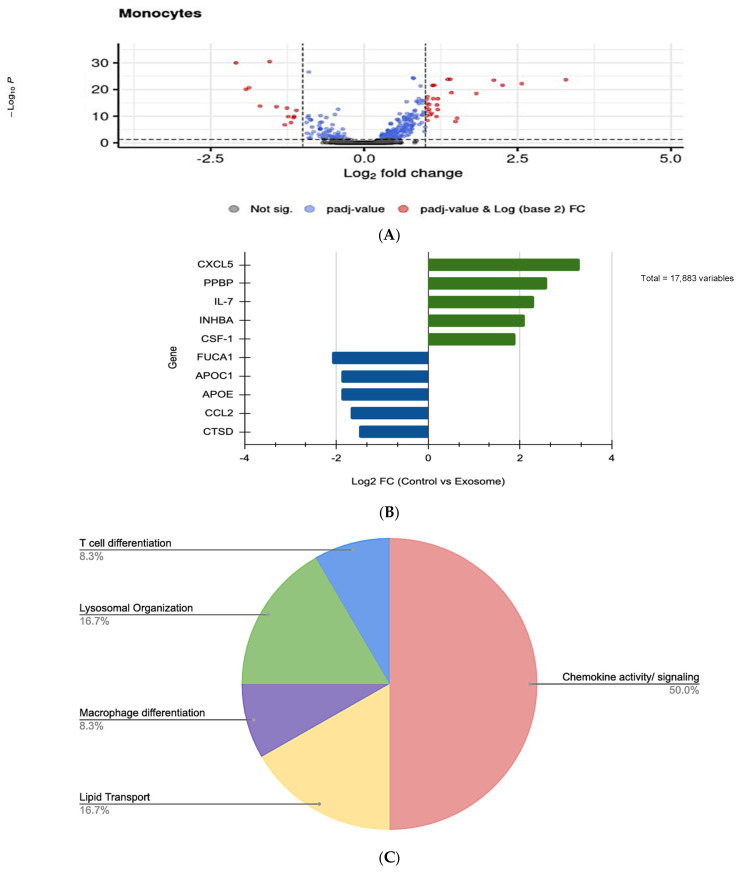
**Top up- and downregulated genes with their functions and GO enrichment analysis.** (**A**) Annotated volcano plot showing differentially expressed genes in monocytes. Labeled genes indicate the most significantly upregulated and downregulated transcripts. (**B**) This plot shows a graph demonstrating the fold change seen in the top up/downregulated genes. Green bars represent top upregulated genes, and blue bars represent top downregulated genes in HAM/TSP PBMC exposed to HTLV-1-derived exosomes compared with Jurkat-derived exosomes. (**C**) Pie chart represents an Enriched GO biological process in top genes. The chart displays the different functions that the top up/downregulated genes have.

To further define the biological processes associated with the observed transcriptional changes, Gene Ontology (GO) enrichment analysis was performed on significantly differentially expressed genes (Figure 3C). Enriched GO biological processes included: (1) chemokine-mediated signaling, (2) immune cell migration and adhesion, (3) macrophage differentiation and polarization, (4) lipid metabolism and transport, (5) lysosomal function and degradation. Key genes driving these pathways included CXCL5, PPBP, IL7R, INHBA, CSF1, FUCA1, APOC1, APOE, and CCL2 (Figure 3A,B). The enrichment of macrophage polarization pathways, alongside both pro-inflammatory and regulatory gene signatures, suggests that exosome-treated monocytes adopt a mixed or hybrid activation state, rather than a strictly classical (M1) or alternative (M2) phenotype.

### 3.5. Exosome-Induced Transcriptional Changes Support Immune Modulation and Functional Reprogramming of Monocytes

**Figure 4:**
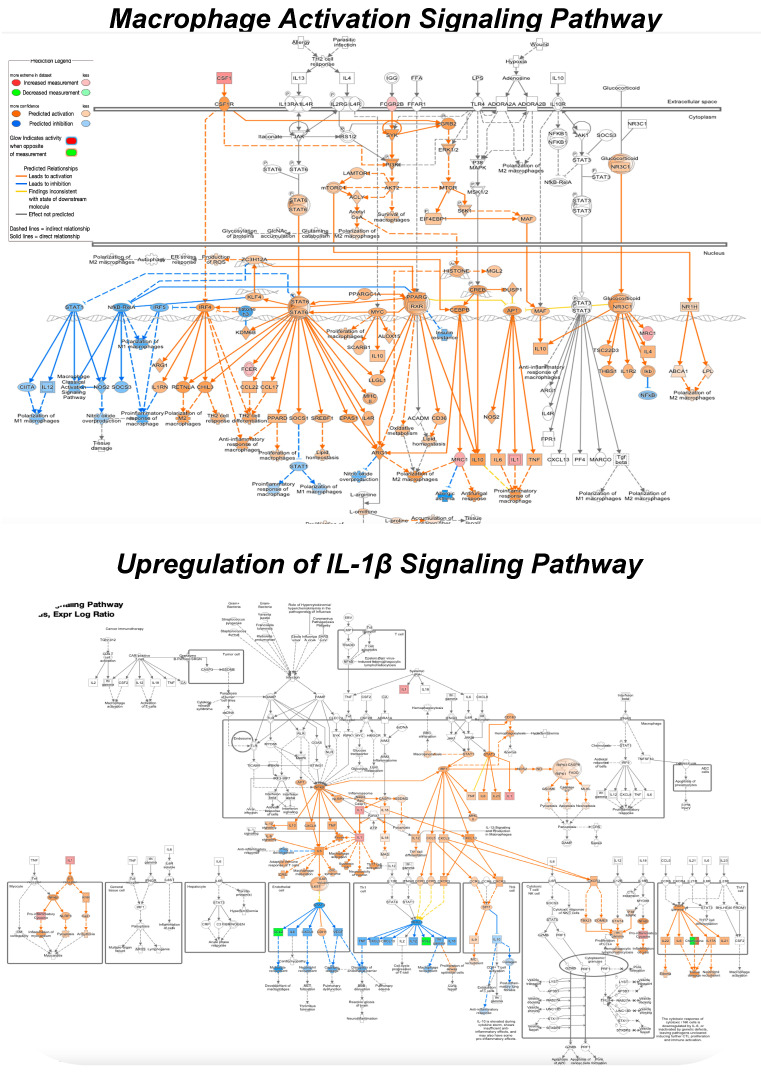
**Ingenuity Pathway Analysis (IPA) of differentially expressed genes (DEGs) in monocytes from HAM/TSP patients without and with exosomes.** Canonical pathway enrichment identified multiple immune-related pathways activated or suppressed in HAM/TSP monocytes treated with HTLV-1-derived exosomes compared with Jurkat-derived exosome controls. Top: The macrophage alternative activation pathway revealed that several of the upregulated genes are involved in monocyte/macrophage differentiation. Those involved in this pathway and upregulated by HTLV-1 are IL-1β, MRC1, CSF1, FCER, and FCGR2B demonstrating a mixed M1/M2 phenotype. Bottom: The Pathogen-Induced Cytokine Storm Signaling pathway highlights the downstream effects of IL-1β that may contribute to viral survival. This pathway suggests that IL-1β is the driving factor in the neuroinflammation seen in the development of HAM/TSP.

To integrate these findings, pathway-level analysis demonstrated that HTLV-1-derived exosomes induce coordinated transcriptional changes affecting immune signaling, metabolism, and cellular function (Figure 4). Upregulated genes were primarily associated with inflammatory signaling, chemokine production, and immune cell recruitment. Whereas downregulated genes were linked to lysosomal degradation, lipid metabolism, and antimicrobial activity. Collectively, these results indicate that HTLV-1-derived exosomes actively reprogram monocyte transcriptional profiles, promoting immune activation while simultaneously altering metabolic and degradative pathways. This dual effect may contribute to persistent inflammation and immune dysregulation observed in HAM/TSP.

## 4. Discussion

The most pronounced transcriptional changes occurred in pathways related to chemokine signaling, lipid metabolism, lysosomal organization, macrophage polarization, and T cell differentiation (Figure 3C). The top upregulated genes, CXCL5, PPBP, IL-7R, INHBA, and CSF-1, were enriched in immune signaling and macrophage-related functions (Figure 4). Specifically, CXCL5 and PPBP promote macrophage recruitment and proliferation, while INHBA and CSF-1 support M2-like polarization [21,22]. Notably, the upregulation of INHBA may indicate increased TGF-β signaling, which has been associated with immunoregulatory and tissue remodeling functions [23]. This suggests that HTLV-1-derived exosomes may contribute to a shift in monocyte phenotype toward a more immunomodulatory state. In contrast, the concurrent upregulation of IL-1β, a key proinflammatory cytokine, highlights the presence of inflammatory signaling. Together, these findings support a mixed polarization profile, rather than a strictly M1 or M2 phenotype, in exosome-treated monocytes. While these transcriptional changes suggest that HTLV-1-derived exosomes may influence monocyte polarization, immune regulation, and inflammatory signaling, the present study is based on transcriptomic analyses and does not establish direct functional consequences. Additional studies will be required to determine whether these pathways contribute to viral persistence, immune evasion, and disease progression in HAM/TSP. IL-7R enhances infected T cell survival by upregulating anti-apoptotic genes [24]. Upregulation of YWHAH may contribute to enhanced cell survival signaling, which could support the persistence of infected or affected cells [25]. Such changes may create a cellular environment favorable for viral maintenance. Collectively, these changes suggest that HTLV-1-derived exosomes skew monocytes toward an anti-inflammatory M2-like state while simultaneously promoting viral persistence by enhancing T cell survival [10,14]. The most downregulated genes, FUCA1, APOC1, APOE, CCL2, and CTSD, further support this immune reprogramming [28]. FUCA1 and CTSD are both involved in glycoprotein degradation and antigen presentation and may impair immune recognition when suppressed [27]. FUCA1 is also involved in preventing viral docking, and by suppressing this gene HTLV-1 may have an easier time infecting surrounding cell. CCL2 (FC 0.31), a chemokine linked to monocyte recruitment and M1 polarization, M2 also decreased, supporting a shift toward immune tolerance. Downregulation of APOC1 (FC 0.27) and APOE (FC 0.27), key in lipid handling, may reflect altered lipid usage favoring viral replication. Overall, our findings suggest that HTLV-1 leverages exosomes to reshape the immune landscape; dampening antigen presentation, promoting an immunoregulatory M2-like environment, and preserving infected T cells.

In our IPA of macrophage alternative activation pathway, four genes were involved that showed significant transcriptional changes: CSF-1, MRC 1, FCER, IL-1β, and FCGR2B (Figure 4). The IPA shows that there is a synergistic effect between CSF-1 and MRC1 because CSF1 enhances expression of MRC1 pushing to M2 development. IL-1β has an opposing role to others in this pathway and may suggest that exosomes facilitate a mixed polarization state or contributes to the chronic inflammatory environment. Interestingly, the upregulation of FCER and FCGR2B suggests an increase in IgE and a decrease in IgG [26,27]. We believe that greater activation of IgE may contribute to an inflammatory environment, suppression of IgG may help viruses evade immune response. These findings further show that HTLV-1-derived exosomes can skew monocyte/macrophage polarization toward a more M2-like, regulatory phenotype, in contrast to the predominantly M1 (proinflammatory) profile observed in HTLV-1 infection without exosomes. This shift may represent a viral strategy to evade immune detection and promote chronic infection. Future directions to determine the full expressivity of M2 macrophages would be to perform HoneyComb technology on cells for IL-10 or TNF-β, which are powerful immunoregulatory cytokines that if increased suggest that exosomes push monocytes to an M2 phenotype. If they are not particularly upregulated, it may point to a more mixed polarization state.

Previous studies have demonstrated substantial cytokine and chemokine dysregulation in HAM/TSP patients, characterized by persistent inflammatory signaling in PBMCs and cerebrospinal fluid, including alterations in chemokines involved in leukocyte trafficking and immune activation. Studies by Carvalho and colleagues, as well as subsequent reviews by Quaresma et al. and Dutartre et al., have highlighted the contribution of dysregulated cytokine networks to chronic neuroinflammation and disease progression in HTLV-1-associated myelopathy [25,26,29]. Our findings complement these observations by suggesting a potential upstream mechanism through which HTLV-1-derived exosomes may influence immune-cell transcriptional programs. In particular, the altered expression of genes involved in cytokine signaling, monocyte differentiation, and immune regulation, including CSF1, INHBA, CXCL5, and CCL2, supports the concept that exosome-mediated communication may contribute to the broader cytokine and chemokine dysregulation previously described in HAM/TSP. Interestingly, while prior studies primarily focused on soluble cytokine and chemokine profiles, the present study provides single-cell transcriptomic evidence that HTLV-1-derived exosomes may reprogram monocytes toward immune-regulatory states associated with altered inflammatory signaling. These findings extend previous observations by linking extracellular vesicle-mediated communication to transcriptional pathways that may contribute to the inflammatory milieu characteristic of HAM/TSP. Notably, IPA identified macrophage differentiation/polarization and chemokine activity/signaling as two of the most significantly enriched functional categories associated with the differentially expressed genes. Key upregulated genes, including CXCL5, PPBP, IL7R, INHBA, and CSF1, were linked to immune-cell recruitment, macrophage activation, and inflammatory signaling pathways. These findings suggest that HTLV-1-derived exosomes not only alter monocyte transcriptional programs but may also reshape the broader cytokine and chemokine environment characteristic of HAM/TSP.

The IPA of the pathogen-induced cytokine signaling pathway illustrates the downstream effect of IL-1β upregulation in Figure 4. IL-1β plays roles in inflammation and is the cytokine responsible for triggering fevers in the body. IL-1β activates IL-1βR which goes on to trigger MAPK and NF-κB to then promote the transcription of TNF, IL-6, IL-8, and GM-CSF [28]. These molecules have various effects throughout the body, but the most profound one is the propagation of the proinflammatory signal through molecules like IL-6 and TNF-a. These molecules have various effects throughout the body, but most notably contribute to the propagation of proinflammatory signaling. In the context of our findings, the upregulation of IL-1β suggests activation of downstream inflammatory pathways, including induction of cytokines such as IL-6 and TNF-α, as well as chemokines like IL-8 and growth factors such as GM-CSF. This supports a role for IL-1β as a key mediator of the inflammatory response observed in exosome-treated monocytes. Overall, IL-1β is essential for the body during an infection to trigger an appropriate response. Over activation of this molecule has been linked to autoimmune disorders, notably rheumatoid arthritis [29]. Correct modulation of IL-1β by the body is crucial for the health of an individual. The observed upregulation of IL-1β suggests that this cytokine may serve as an important mediator of exosome-induced inflammatory responses in HAM/TSP. Given its central position within cytokine signaling networks and its ability to induce downstream inflammatory mediators including TNF-α, IL-6, and IL-8, IL-1β may contribute to the chronic neuroinflammatory environment associated with disease progression.

Overall, our findings demonstrate that HTLV-derived exosomes reprogram monocytes in HAM/TSP patient specimens, inducing significant transcriptional changes that promote a mixed M1/M2-like phenotype rather than the classically proinflammatory M1 state typically associated with viral infection. Exosome-mediated signaling appears to contribute to immune evasion and sustained neuroinflammation, highlighting a previously underappreciated role for monocytes in HAM/TSP pathogenesis. Future studies should functionally validate the identified genes and pathways, particularly those involved in M2 polarization and IL-1β-mediated cytokine signaling, using protein-level analyses, cytokine profiling, and established polarization markers (e.g., IL-10, TNF-β). Collectively, these findings suggest that targeting exosome production, release, or uptake may represent a novel therapeutic strategy to disrupt viral communication, restore immune balance, and mitigate neuroinflammation in HAM/TSP.

## Figures and Tables

**Table 1:** **List of up and down regulated genes in the descending order of fold change with significance**.

Gene	Gene Description	Linear FC	*p*-Values
Up Regulated			
CXCL5	C-X-C motif chemokine ligand 5	9.85	1.25 × 10^−28^
PPBP	pro-platelet basic protein	6.06	3.47 × 10^−27^
IL7R	interleukin 7 receptor	4.93	1.33 × 10^−26^
INHBA	inhibin subunit beta A	4.29	1.78 × 10^−28^
CSF1	colony stimulating factor 1	3.48	1.67 × 10^−23^
CCL24	C-C motif chemokine ligand 24	2.83	3.22 × 10^−14^
IL1B	interleukin 1 beta	2.83	5.97 × 10^−13^
ARRDC4	arrestin domain containing 4	2.64	8.10 × 10^−24^
MRC1	mannose receptor C-type 1	2.64	6.99 × 10^−29^
CDKN1A	cyclin dependent kinase inhibitor 1A	2.64	8.19 × 10^−29^
SPN	sialophorin	2.30	1.66 × 10^−21^
SDC2	syndecan 2	2.30	1.70 × 10^−17^
MMP9	matrix metallopeptidase 9	2.30	8.01 × 10^−15^
CLEC4A	C-type lectin domain family 4 member A	2.14	1.29 × 10^−26^
GSN	granulin precursor	2.14	1.39 × 10^−21^
FCER2	Fc epsilon receptor II	2.14	1.66 × 10^−26^
EMP1	epithelial membrane protein 1	2.14	4.75 × 10^−16^
SLC44A1	solute carrier family 44-member 1	2.14	1.62 × 10^−24^
SLC11A1	solute carrier family 11-member 1	2.14	2.46 × 10^−19^
GPAT3	glycerol-3-phosphate acyltransferase 3	2.14	1.29 × 10^−17^
FCGR2B	Fc gamma receptor IIb	2.00	7.43 × 10^−17^
PTPRE	protein tyrosine phosphatase receptor E	2.00	2.90 × 10^−22^
KCNK6	potassium two pore domain channel subfamily K member 6	2.00	2.03 × 10^−13^
ITGAM	integrin subunit alpha M	2.00	9.62 × 10^−20^
SERPINE1	serpin family E member 1	2.00	3.77 × 10^−15^
FBP1	fructose-bisphosphatase 1	2.00	3.47 × 10^−21^
TXNIP	thioredoxin interacting protein	2.00	2.65 × 10^−18^
**Down Regulated**			
FUCA1	alpha-L-fucosidase 1	0.233	5.68 × 10^−35^
APOC1	apolipoprotein C1	0.268	4.72 × 10^−25^
APOE	apolipoprotein E	0.268	1.18 × 10^−25^
CCL2	C-C motif chemokine ligand 2	0.308	8.71 × 10^−19^
CTSD	cathepsin D	0.354	1.69 × 10^−35^
GPNMB	glycoprotein nmb	0.379	1.53 × 10^−18^
SLAMF7	SLAM family member 7	0.406	9.75 × 10^−12^
FYB1	FYN binding protein 1	0.406	5.04 × 10^−18^
SOD2	superoxide dismutase 2	0.435	8.31 × 10^−15^
ATF5	activating transcription factor 5	0.435	1.44 × 10^−12^
CYP27A1	cytochrome P450 family 27 subfamily A member 1	0.467	2.01 × 10^−14^
PLXNC1	plexin C1	0.467	6.69 × 10^−15^
LYZ	lysozyme	0.467	3.76 × 10^−17^

## Data Availability

The data presented in this study are openly available in the NCBI Gene Expression Omnibus (GEO) repository under accession number GSE308157.

## Supplementary Materials

The following supporting information can be downloaded at: https://www.mdpi.com/article/10.3390/pathogens15070704/s1, Figure S1: Quality Control and cell type annotation of single-cell transcriptomic data. (A) Violin plots showing quality control (QC) metrics before (left) and after (right) filtering of single cells from HAM/TSP with and without samples. Cells were retained if they expressed <4500 genes (to remove multiples), >100 genes (to eliminate empty droplets), <10% mitochondrial gene content (to exclude dying cells), and <45% ribosomal or <20% hemoglobin content (to minimize noise). (B) Table indicates the total cells assigned to each annotated immune-cell population in the Jurkat-derived control exosome and HTLV-1-derived exosome treatment groups. (C) UMAP showing clustering of major immune cell types identified using canonical marker expression and SingleR-based annotation aligned with the Human Cell Atlas. (D) Stacked bar plot indicating the proportion of each immune cell type across HAM/TSP and AC samples, revealing compositional differences in PBMC subsets.
