# Supplementary figures and images for: HTLV-1-Derived Exosomes Drive Transcriptional Reprogramming of Monocytes Toward a Mixed M1/M2 Phenotype in HAM/TSP
Source: Pathogens. 2026 Jul 3;15(7):704. doi: 10.3390/pathogens15070704 (PMC13414601; doi:10.3390/pathogens15070704)

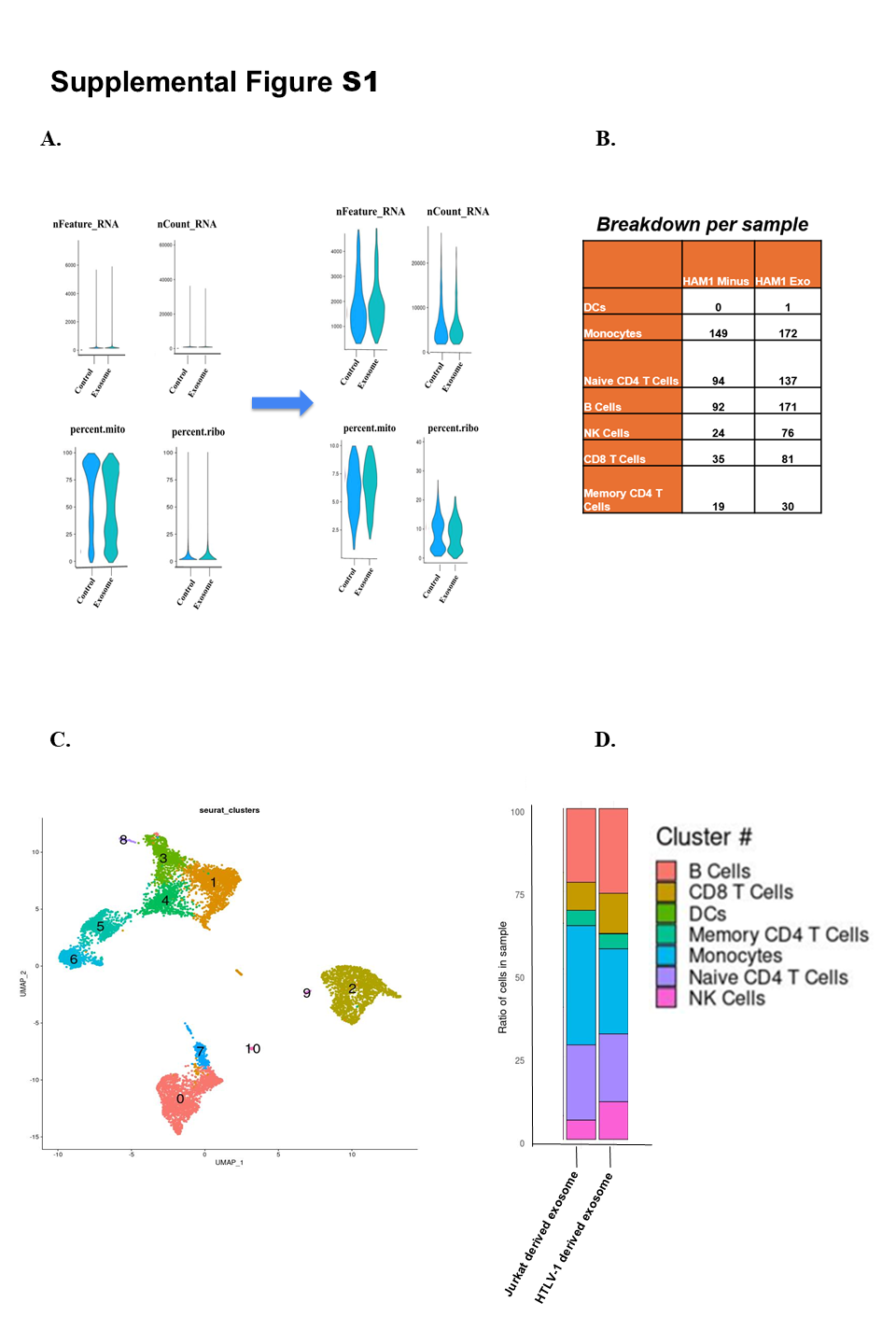

Supplement: Supplementary file 1 [file pathogens-15-00704-s001.zip › pathogens-4272957-supplementary.png]
